# Preliminary Study on Pasta Samples Characterized in Antioxidant Compounds and Their Biological Activity on Kidney Cells

**DOI:** 10.3390/nu13041131

**Published:** 2021-03-30

**Authors:** Federico Di Marco, Francesco Trevisani, Pamela Vignolini, Silvia Urciuoli, Andrea Salonia, Francesco Montorsi, Annalisa Romani, Riccardo Vago, Arianna Bettiga

**Affiliations:** 1 Division of Experimental Oncology, Urological Research Institute (URI), IRCCS San Raffaele Scientific Institute, 20132 Milan, Italy; federico.dimarco91@gmail.com (F.D.M.); trevisani.francesco@hsr.it (F.T.); salonia.andrea@hsr.it (A.S.); montorsi.francesco@hsr.it (F.M.); 2 PHYTOLAB (Pharmaceutical, Cosmetic, Food Supplement, Technology and Analysis), DiSIA, University of Florence, 50019 Sesto Fiorentino, Italy; pamela.vignolini@unifi.it (P.V.); silvia.urciuoli@gmail.com (S.U.); annalisa.romani@unifi.it (A.R.); 3 Department of Urology, Università Vita-Salute San Raffaele, 20132 Milan, Italy

**Keywords:** pasta, Mediterranean diet, polyphenols, carotenoids, antioxidant compounds, kidney health, HPLC/DAD analyses

## Abstract

Pasta is one of the basic foods of the Mediterranean diet and for this reason it was chosen for this study to evaluate its antioxidant properties. Three types of pasta were selected: buckwheat, rye and egg pasta. Qualitative–quantitative characterization analyses were carried out by HPLC-DAD to identify antioxidant compounds. The data showed the presence of carotenoids such as lutein and polyphenols such as indoleacetic acid, (carotenoids from 0.08 to 0.16 mg/100 g, polyphenols from 3.7 to 7.4 mg/100 g). To assess the effect of the detected metabolites, in vitro experimentation was carried out on kidney cells models: HEK-293 and MDCK. Standards of β-carotene, indoleacetic acid and caffeic acid, hydroalcoholic and carotenoid-enriched extracts from samples of pasta were tested in presence of antioxidant agent to determine viability variations. β-carotene and indoleacetic acid standards exerted a protective effect on HEK-293 cells while no effect was detected on MDCK. The concentrations tested are likely in the range of those reached in body after the consumption of a standard pasta meal. Carotenoid-enriched extracts and hydroalcoholic extracts showed different effects, observing rescues for rye pasta hydroalcoholic extract and buckwheat pasta carotenoid-enriched extract, while egg pasta showed milder dose depending effects assuming pro-oxidant behavior at high concentrations. The preliminary results suggest behaviors to be traced back to the whole phytocomplexes respect to single molecules and need further investigations.

## 1. Introduction

Despite that there is no unique definition of the Mediterranean diet—due to the variations and adaptations in culinary traditions not only around the Mediterranean area but also around the world [1]—the Mediterranean diet may be thought of as having several components that meet important criteria for a healthy diet: low content of saturated fatty acids, high content of composite carbohydrates, dietary fiber and antioxidant molecules, and abundance in vegetables.

It is difficult to establish which foods, or active ingredients therein contained, of the Mediterranean diet are most responsible for the health benefits; accumulating data suggest that its main benefits lie in the combination of the complex and wide variety of different nutrients interacting synergistically and additively [2].

In recent years, the growing literature (observational studies and randomized control trials) has demonstrated the health benefits associated with the Mediterranean diet, reducing risk of developing multiple chronic diseases as cardiovascular diseases [3,4,5], diabetes [6,7,8,9,10], cancer [11,12], obesity [13,14,15] or cognitive health [16,17,18,19] and increasing life expectancy [20].

Improvements in blood pressure [21], lipid profile [22,23], insulin resistance [24], and protection against oxidative stress, inflammation, platelet aggregation and endothelial dysfunction [25,26,27,28] seem to be the mechanisms of action responsible for the beneficial effects on the general health. All these biological effects promoted by the adherence to the Mediterranean lifestyle could be relevant not only for the above-mentioned chronic diseases, but also for the prevention of renal decay in general population. Chronic kidney disease (CKD) represents a global public health problem, affecting over 750 million persons worldwide and acts often silent and unhindered thanks to its asymptomatic clinical presentation. Different lines of evidence have underlined the strong relationship between Mediterranean diet and renal preservation [29].

Considering all the above-mentioned effects as risk factors for the decline of the renal function, the kidney can be considered one of the primary targets.

A recent meta-analysis of cohort studies has shown that plant-based diets such as the Mediterranean diet or dietary approach to stop hypertension (DASH) diet was associated with a lower risk of incident CKD and albuminuria in the general population [30]. One explanation could be that fruit and vegetables contain bioactive compounds protecting against inflammation and endothelial dysfunction that promote dynamic changes of filtration fraction, resulting in a progressive reduction of the glomerular filtration rate, extracellular fluid volume expansion, abnormal ion balance, and renal hypoxia, ultimately leading to loss kidney function [31,32,33]. Moreover, the increase of vegetable, fruit, cereal and legumes consumption may have led to a decrease in animal protein consumption positively influencing acid-base balance and glomerular hemodynamics of the kidney, protecting it to glomerular sclerosis and loss of function [34,35,36,37].

Despite this, there are several studies with contrasting data for the impact of a high protein diet on renal function decline in the general population [38,39,40,41] growing evidence suggest that the protein source plays an important role to preservation of renal function, and that a shift from animal to plant source of protein might be beneficial [42,43].

Pasta is one of the most consumed foods in the world and is one of the staple foods of the Mediterranean diet. Traditionally, Italy is the main producer and leader of the pasta consumption, even if pasta is consumed worldwide for low cost, palatability, and the longer shelf life than other bakery products [44]. Pasta is a good source of carbohydrates and energy (100 g of cooked pasta contains about 31 g of carbohydrates and about 158 kcal) but it is typically low in lipids, proteins, phosphorus and potassium that are nutrient components restricted in the most renal diets for kidney disease (available online at https://fdc.nal.usda.gov, accessed on 1 December 2020). Naturally enriched wheat pastas, which represent the majority of commercially available pastas, also offer good levels of thiamin, riboflavin, niacin, folate, iron, and selenium, as well as polyphenols. Furthermore, pasta is always consumed in combination with other food items that could be considered as a stronger source of polyphenols, such as olive oil and vegetables. Pasta is usually produced with durum wheat semolina, due to its excellent rheological properties, the superior color of the pasta, the quality of cooking and the acceptance by the consumer, but lately pasta can also be produced starting from other cereals such as rice, buckwheat, rye, spelled.

In recent years, consumers have become more aware that a balanced diet, such as the Mediterranean one, can positively affect health, which is why the food industry tries to meet these demands by producing quality and functional foods and food ingredients.

Despite fortified foods have been produce for healthy purpose, people could be induced to assume them instead of a balanced diet. This is a so important topic to prompt the FDA to publish an ad hoc document entitled “FDA’s fortification policy” to give an answer on one side to the food industry and on the other to the academic world [44].

Balanced, adequate and varied diet is an important step towards a happy and healthy lifestyle without the risk of overloading with some added component contained in fortified food. Precisely for this reason non-traditional cereals and pseudo-cereals have been rediscovered which have paved the way for their use as functional food ingredients as they possess some nutritional and functional qualities that are absent or lacking in traditional cereals. Buckwheat and rye are a rich source of phytochemicals such as polyphenols, compounds that are strongly correlated with antioxidant activities [45,46].

Launched in March 2005, the MOLI-SANI Project involved about 25,000 citizens residing in Molise (Italy), to evaluate environmental and genetic factors underlying cardiovascular diseases, tumors and neurodegenerative diseases. According to a study from the MOLI-SANI project, pasta intake is associated with a lower BMI and lower levels of central obesity. Many studies have confirmed that high calorie diets lead to weight gain and not a diet that includes carbohydrates. In fact, if the portion of pasta is correct and the sauce is not too caloric, a plate of pasta can have a caloric content that respects the daily energy requirement [47]. Pasta is held in high regard as its characteristics have been associated not only with body weight control, but also with several positive health properties [48]. A randomized, controlled study conducted on healthy subjects showed that rye-based products improve glycemic regulation, increase intestinal hormones involved in the regulation of appetite and metabolism. Rye reduces postprandial appetite levels by reducing the desire to eat at subsequent meals, increasing satiety and reducing hunger by increasing the satiety hormones GLP-1 (glucagon-like peptide-1) and peptide YY (PYY). A lower postprandial glucose response was observed in a group of people consuming rye-based foods compared to a group of subjects consuming refined wheat. This happens because rye is a low glycemic index cereal that can also be used in diabetic patients [49]. Rye can improve the plasma lipid profile, in fact the intake of whole rye is inversely associated with the concentration of LDL cholesterol, the LDL/HDL ratio and the concentration of triglycerides. This effect is due to the presence of fibers and β-glucans [50].

Pasta is well known as a source of carbohydrates, but it can also contain minor compounds, such as carotenoids [51], especially lutein, and other antioxidant species such as polyphenols. These bioactive molecules can be enriched in different types of pasta, depending on the raw materials. For instance, the carotenoids content of durum wheat is higher than that of bread wheat [52] and the level of antioxidants can be increased by incorporating bran fraction and entire kernel of durum wheat to pasta products [53]. Buckwheat is a substantial source of phenolic compounds, vitamins and essential amino acids [54], while the addition of egg in the preparation of the pasta conveys carotenoids and lutein. Carotenoids participate in prevention activity, especially in inflammation syndrome and their role is due to their antioxidant properties [55] against free radicals and singlet oxygen [56]. Pasta contains different types of antioxidant molecules and for the frequent consumers, large consumption can be considered an important source of such healthy compounds.

Here we show that different types of pasta namely buckwheat, rye and egg pasta, contain various amounts of antioxidant compounds, which have a role to improve renal cell viability at a steady state or upon the induction of oxidative stress.

## 2. Materials and Methods

### 2.1. Samples

Three samples of cooked pasta were analyzed and tested: one sample of wholemeal buckwheat pasta (1), one sample of wholemeal rye pasta (2) and one sample of egg pasta of stone-ground durum wheat flour (3).

### 2.2. Pasta Samples Preparation

Totals of 50 g of whole meal buckwheat pasta or whole meal rye pasta were cooked in 500 mL of water for 7 min; 50 g of egg pasta of stone-ground durum wheat flour were cooked in 500 mL of water for 1 min, as indicated by the manufacturer.

To obtain extracts enriched in carotenoids, 10 g of cooked pasta were dissolved in 100 mL acetone, cold sonicated for 30 min. The sample was centrifuged for 5 min at 5000 rpm, the supernatant has been dry evaporated with a rotary evaporator and the residue was dissolved in acetone or DMSO depending on the downstream analysis. To obtain extracts enriched in polyphenols, 10 g of each sample of cooked pasta were dissolved in 50 mL of 70:30 EtOH/H_2_O at pH 3.2. The samples were shaken for 24 h, centrifuged for 5 min at 1400 rpm. The sample was centrifuged for 5 min at 5000 rpm and the supernatant was collected and analyzed in HPLC-DAD, while an aliquot was dry evaporated, and the residue dissolved in DMSO for cell treatment.

### 2.3. HPLC-DA-MS Analysis

Quali-quantitative analyses of carotenoids and polyphenols were carried out using an HP 1100 liquid chromatography equipped with a DAD detector and managed by an HP 9000 workstation (Agilent Technologies, Palo Alto, CA, USA) and linked to a mass spectrometer with an API/electrospray interface (Agilent Technologies). The mass spectrometer operating conditions were as follows: gas temperature, 350 °C; nitrogen flow rate, 11.0 L/min; nebulizer pressure, 40 psi; quadrupole temperature, 100 °C; and capillary voltage, 4000 V. The mass spectrometer was operated in positive and negative modes at 80–180 eV.

Compounds were separated using a 250 × 4.6 mm i.d, 5 µm LUNA C18 column (Phenomenex, Torrance, CA, USA). UV/Vis spectra were recorded in the 190–600 nm range and the chromatograms were acquired at 250, 280, 330, 350 and 450 nm. The samples were analyzed by gradient elution at a flow rate of 0.8 mL/min. The mobile phase for carotenoids was a multistep linear solvent gradient system (solvent A: acetone, solvent B: H_2_O, pH 3.2 by formic acid), starting from 80% acetone up to 100% in 30 min. polyphenols were eluted using the following gradient: from 90% H_2_O (adjusted to pH 3.2 by formic acid) to 100% CH_3_CN in 40 min. All solvents used were of HPLC grade purity (BDH Laboratory Supplies, Poole, UK).

### 2.4. Quantitative Analysis

Quantification of individual polyphenolic compounds was directly performed by HPLC-DAD using a five-point regression curve (R^2^ ≥ 0.998) in the range of 0–30 µg on the basis of authentic standards. In particular, flavonols were determined at 350 nm using quercetin 3-O-glucoside as reference compound while caffeic acid derivatives were determined at 330 nm using clorogenic acid as reference compound and indoleacetic acid derivative at 280 nm using 3 indoleacetic acid (Sigma-Aldrich, St. Louis, MO, USA). Carotenoids were determined at 450 nm using β beta-carotene as reference compound (Extrasynthese, Lione, Francia). In all cases, actual concentrations of the derivatives were calculated after applying corrections for differences in molecular weight.

### 2.5. Total Phenolic Content

The total phenolic content was determined using the Folin–Ciocalteu method, described by Singleton et al. [57] and slightly modified according to Dewanto et al. [58]. To 125 µL of the suitably diluted sample extract, 0.5 mL of deionized water and 125 µL of the Folin–Ciocalteu reagent were added. The mixture was kept for 6 min and then 1.25 mL of a 7% aqueous Na_2_CO_3_ solution were added. The final volume was adjusted to 3 mL with water. After 90 min, the absorption was measured at 760 nm against water as a blank. The amount of total phenolics is expressed as gallic acid equivalents (GAE, mg gallic acid/100 g sample) through the calibration curve of gallic acid. The calibration curve ranged from 20 to 500 µg/mL (R^2^ = 0.9969) [57,58].

### 2.6. Cell Culture

Human Embryonic Kidney 293 (HEK-293) cells and Madin–Darby canine kidney (MDCK) cells were cultured in DMEM supplemented with GlutaMAX, 10% (*v*/*v*), fetal bovine serum, 100 U/mL penicillin and100 µg/mL streptomycin at 37 °C with 5% CO_2_.

### 2.7. MTT Assay

5000 cells/well MDCK and HEK-293 cells were seeded in 96 well plates and allowed to adhere for 24 h. For viability studies, cells were incubated for 24 h to 72 h with beta-carotene, indole-3-acetic acid, caffeic acid (in DMSO) and pasta extracts alone or in presence of magnesium monoperoxyphtalate (MMPP, Sigma-Aldrich, St. Louis, MO, USA). DMSO in the medium at equal concentrations to those used for the tested compounds was used for untreated samples. The tested concentration for the standards were ranging from 2 pM to 10 µM, while for MMPP from 0.001 to 0.05 mg/mL. Cell viability were measured 3-(4,5-dimethylthiazol-2-yl)-2,5-diphenyl tetrazolium bromide (MTT, Sigma-Aldrich, St. Louis, MO, USA). The plates were incubated at 37 °C for 1 h and then the Formazan produced by the MTT reduction was solubilized in DMSO. Absorbance was determined on a micro plate reader (Mithras LB 940-Berthold) at 570 nm. The percentage of cell viability was calculated using the ratio Abs_TEST_/Abs_CTRL_.

### 2.8. Statistical Analysis

Each experiment was performed in quadruplicate and repeated at least three times. Differences in viability percentages were assessed using paired Student’s t test and two-way ANOVA followed by a post-hoc test with Holm’s correction for multiple comparison over concentrations metabolites (*p* < 0.05). Results were expressed as mean ± standard error and the analysis were performed by R-Studio environment [59] for R version 3.6.3 [60] using the package “Tidyverse” [61].

## 3. Results and Discussion

### 3.1. Determination of the Chemical Quality of Pasta Samples

The nutritional information of a food allows making informed food and dietary choices. Pasta is traditionally prepared from semolina but even with other cereals, all contain starch as a principal constituent followed by protein, fat, vitamins, minerals and bioactive compounds [62]. In Table S1 are reported the nutritional data.

To compare the nutritional values of several types of pasta enriched in bioactive compounds due to the different source of cereals or to the supplementation of specific ingredients, we analyzed buckwheat pasta, rye pasta and egg pasta. In order to obtain extracts enriched in the various subclasses of compounds present in the pasta samples, both hydroalcoholic and acetone extractions were carried out and analyzed. Individual polyphenols were tentatively identified using data from HPLC-DAD-MS analysis by comparison and combination of their retention times and mass spectrometry and UV spectra (see Figures S1–S5 for same example of MS spectra) and comparing results with standards (kaempferol, quercetin, rutin and ferulic acid) and previous bibliographic works. In particular, in hydroalcoholic extracts, flavonoids, caffeic and indoleacetic acid derivatives were identified (Table 1): the indoleacetic acid derivative is the main compound as described before even for semolina. [63]. Considering relative compositions, buckwheat pasta showed higher levels of flavonoids, in particular quercetin derivatives are the main flavonols as previously reported in buckwheat [64,65], and negligible presence of caffeic acid derivatives; rye pasta showed higher levels of caffeic acid derivatives, in particular ferulic acid [66] and low content of flavonoids; egg pasta presented only low concentration of flavonoids (apigenin derivative [67]) and no presence of caffeic acid derivatives.

**Figure 1:**
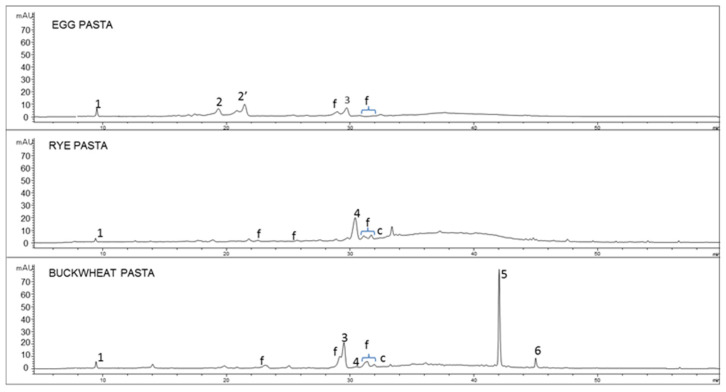
Chromatographic profile (Relative abundance as y axis vs. Retention time as x axis) acquired by HPLC-DAD (350 nm) of the hydroalcholic extracts of cooked buckwheat pasta, rye pasta, egg pasta. Identified compounds: 1 = indoleacetic acid derivative, 2,2′ = apigenin diglicosides, 3 = rutin, 4 = ferulic acid, 5 = quercetin, 6 = kaempferol, f = flavonoids.

As an example, the chromatographic profiles of cooked pasta samples, recorded at 350 nm, are presented in Figure 1. The figure reveals the qualitative composition of the samples analyzed.

**Figure 2:**
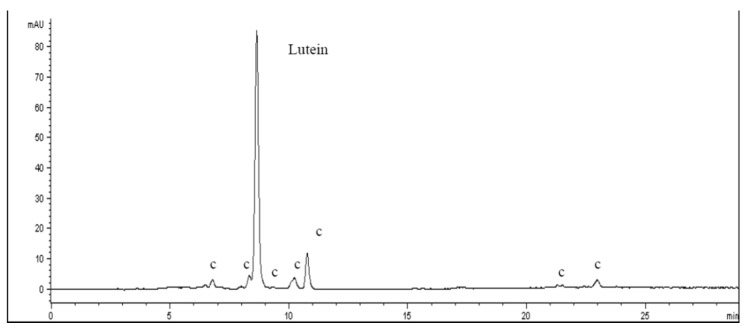
Chromatographic profile (Relative abundance as y axis vs. Retention time as x axis) at 450 nm of a carotenoid acetone fraction of egg cooked pasta extract. Identified compounds: Lutein, c = carotenoid derivatives.

Even carotenoids were pointed out in egg pasta and buckwheat pasta, and Figure 2 shows the HPLC-DAD chromatogram (450 nm) of the acetone extract of cooked egg pasta sample.

Lutein was the main carotenoid identified in the acetone extracts, with similar relative content with respect to the overall carotenoids species, as shown in Table 2, in line with previous studies [68]. Carotenoids were not detected in rye pasta.

The presence of antioxidant secondary metabolites has been evaluated in the pasta samples as well (Table 3). The total antioxidant capacity of biocomponents, likely due to the activity of phenol/polyphenol was assessed and the amount of such molecules was deduced.

Buckwheat pasta sample has higher values than other pasta’s samples in terms of total phenolic content.

### 3.2. Effect of Indole-3-Acetic Acid, β-Carotene and Caffeic Acid on Cells Viability

**Figure 3:**
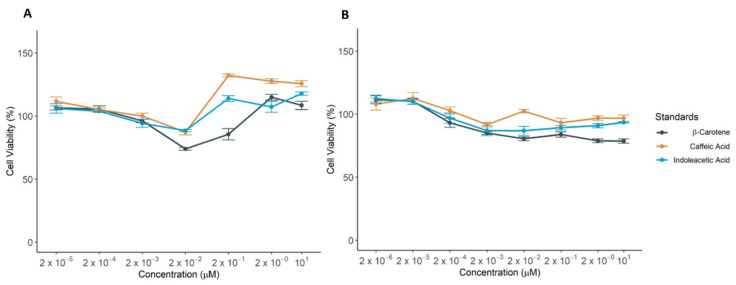
Detection cell viability of HEK-293 (**A**) and MDCK (**B**) incubated with increasing amount of indoleacetic acid, β-carotene and caffeic acid for 24 (**B**) or 72 h (**A**). Reported values correspond to mean of cell viability with standard error over three biological replicates. The percentage of cell viability was calculated using the ratio Abs_TEST_/Abs_CTRL_.

Mediterranean diet has been recognized as a source of anti-oxidant molecules, which protect the organism against oxidative stress and support the healthy status [2]. Therefore, we wondered whether the biomolecules detected in our pasta samples can affect cell viability and at which extent. We used HEK-293 and MDCK healthy kidney cells to determine the activity of indoleacetic acid, β-carotene and caffeic acid standard compounds. The kidney is especially susceptible to oxidative stress and the accumulation of free radicals leads to renal impaired function [69]. Cells were incubated with scalar concentration of the standard compounds and the cell viability was measured at different time points (Figure 3 and Figure S6).

They were found not to exert any toxic effect on HEK-293 cells for all the tested concentrations at times of incubation. Only β-carotene reduced cell viability in a u-shape manner on HEK-293 cells and in a dose-dependent manner in MDCK cells (Figure 3). Remarkably, the concentrations likely reached in body after the consumption of a standard pasta meal (usually 80 gr) are in the range of the lowest tested (2 × 10^−5^–2 × 10^−4^ µM, [70,71]), which provided a weak enhancement of the viability on both cell lines. On the other hand, at higher concentrations above 0.2 μM, which can be obtained only pharmacologically, caffeic acid showed a significant improvement of the cell viability, while indoleacetic acid and β-carotene showed comparable effects along the increase of concentration (2–20 µM) in HEK-293 cells along the time.

### 3.3. Effect of Indole-3-Acetic Acid, β-Carotene and Caffeic Acid on MMPP-Treated Cells

**Figure 4:**
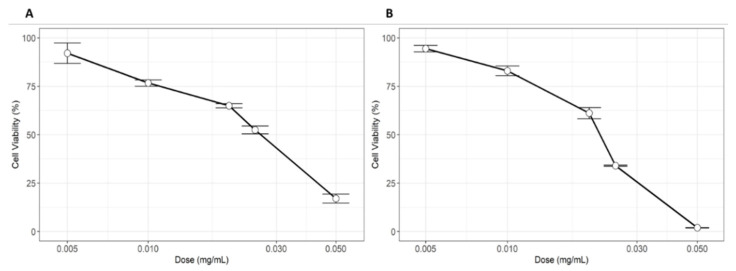
Effect of magnesium monoperoxyphtalate (MMPP) on cell viability of HEK-293 (**A**) and MDCK (**B**) after 24 (**B**) or 72 (**A**) hours incubation.

We next wondered if indoleacetic acid, β-carotene and caffeic acid can counteract a stress induced by the oxidizing agent MMPP. First, we tested the MMPP effect by exposing HEK-293 and MDCK cells to serial dilutions (0.005–0.05 mg/mL) to the drug and the cell viability was determined. A dose-dependent decrease following MMPP treatment was noticed and the IC50 was comparable in the two cell lines tested. After incubation with 20–25 µg/mL MMPP the cell viability was significantly decreased to around 50–60% (Figure 4) and the half maximal inhibitory concentration (IC_50_) was calculated to be 26 µg/mL for HEK-293 and 22 µg/mL for MDCK.

**Figure 5:**
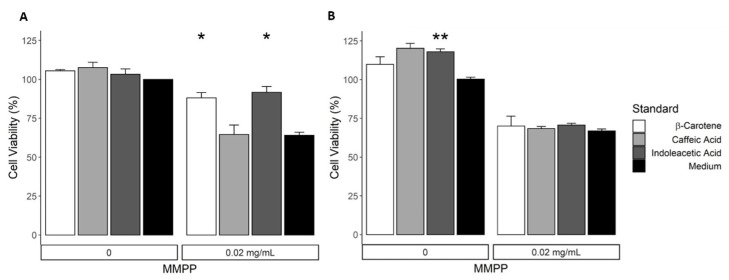
Effect of indoleacetic acid, β-carotene and caffeic acid on the cell viability of HEK-293 (**A**) and MDCK (**B**) in the absence or presence of MMPP after 24 (**B**) or 72 (**A**) hours incubation. Reported values correspond to mean of cell viability with standard error over three biological replicates. The percentage of cell viability was calculated using the ratio Abs_TEST_/Abs_CTRL_. * *p* < 0.05; ** *p* < 0.01.

Then, we tested the protective activity of our anti-oxidant molecules on MMPP-treated cells, by supplementing the cell media with 2 × 10^−5^ nM indoleacetic, β-carotene or caffeic acid. A slightly positive effect on cell viability of both HEK-293 and MDCK was confirmed by employing the standards as such at the dose likely derived from a pasta portion (Figure 5).

As expected, MMPP reduced the call viability to 60–65%; the treatment with indoleacetic acid or β-carotene counteract the MMPP effect and rescued the cell viability by 30% (*p* < 0.001) and 27% (*p* > 0.01) respectively on HEK-293 cells, while caffeic acid did not exert any significant effect. In the presence of an oxidant agent, beta-carotene and indoleacetic acid showed a protective effect on the cells at a low concentration, whereas caffeic acid did not counteract oxidative stress at the concentration needed to enhance cell viability. Those positive effects are likely due to the antioxidant properties of indoleacetic acid and carotenoids that can significantly counterbalance the MMPP-mediated cytotoxicity at the dose used in this experimental condition. Nevertheless, no significant rescue was observed on MMPP-treated MDCK cells, suggesting that, even of standards alone displayed a significantly positive effect on cell viability, they are not sufficient to tackle the MMPP-induced oxidative stress. Embryonal HEK-293 cells seem to be more prompt to arrange a defense line against stress than adult MDCK, presumably due to additional cellular redox homeostasis systems.

### 3.4. Effect of the Pasta-Derived Carotenoid-Enriched Fraction on MMPP-Induced Cytotoxicity

**Figure 6:**
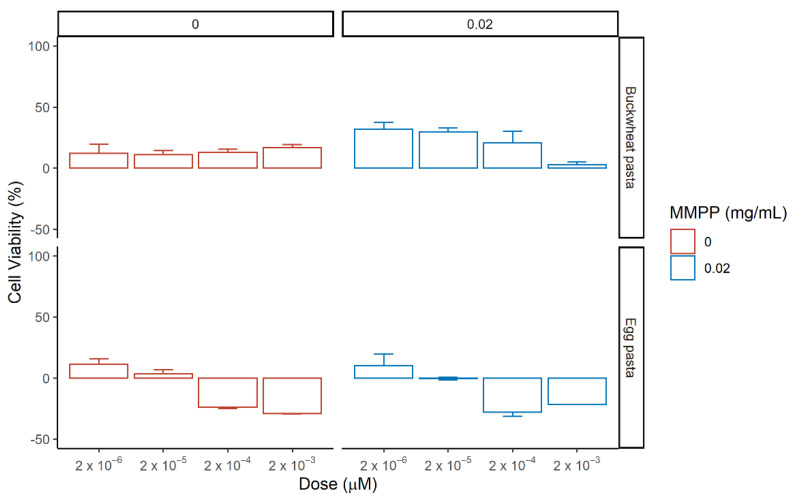
Effect of the pasta-derived carotenoid-enriched fractions on the cell viability of HEK-293 in the absence (left, red) or presence (right, blue) of MMPP after 72 h incubation. Reported values correspond to mean of the difference in cell viability between tested phytocomplexes and reference (medium in red, and MMPP in blue) with standard error over three biological replicates. The percentage of cell viability was calculated using the ratio Abs_TEST_/Abs_CTRL_.

To evaluate the effects of the carotenoid-enriched fractions derived from the acetone extraction of buckwheat pasta and egg pasta on renal cells in the presence of oxidative stress, HEK-293 cells were incubated with different concentrations of extracts ranking from 2 × 10^−6^ µM to 2 × 10^−3^ µM in presence of MMPP for 72 h (Figure 6 and Table S2). The concentration used refers to the of β-carotene concentration detected in the extract (Table 2). The lutein content ranged from 1.5 × 10^−6^ µM to 1.5 × 10^−3^ µM in Buckwheat pasta and from 1.34 × 10^−6^ µM to 1.34 × 10^−3^ µM in egg pasta.

In both acetone extracts, the presence of natural phytocomplexes, showed different effects with respect to the single standard, resulting more active at lower concentrations. As the concentration of extracts from egg pasta increases, a reduction of cell viability was observed. The lowest concentration of carotenoid extracts (2 × 10^−6^ µM) induced a modest positive effect alone or in the presence of MMPP on HEK-293 cells. Buckwheat pasta extract generated an enhancement of cell viability as the extract alone, but also in the presence of MMPP ranging from 32 ± 5% at lowest concentration to 21 ± 9% for 2 × 10^−4^ µM.

**Figure 7:**
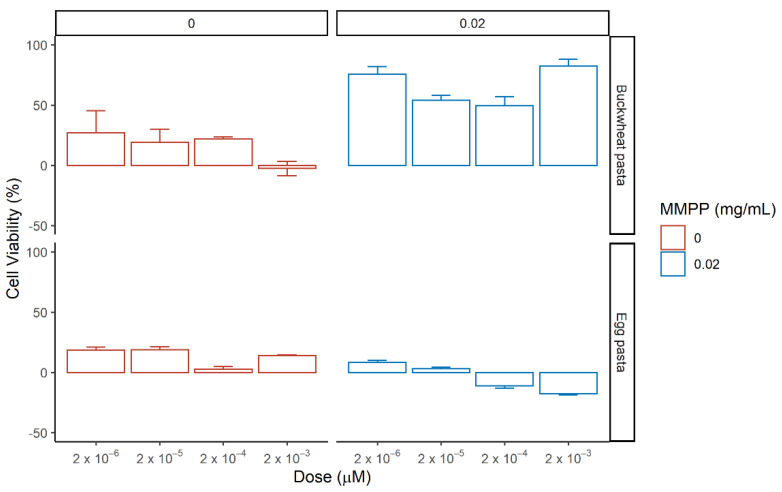
Effect of the pasta-derived carotenoid-enriched fractions on the cell viability of Madin–Darby canine kidney (MDCK) in the absence (left, red) or presence (right, blue) of MMPP after 24 h incubation. Reported values correspond to mean of the difference in cell viability between tested phytocomplexes and reference (medium in red, and MMPP in blue) with standard error over three biological replicates. The percentage of cell viability was calculated using the ratio Abs_TEST_/Abs_CTRL_.

The activity of acetone extracts was assessed also on MDCK cells in presence or absence of MMPP and compared with that obtained in HEK-293 cells (Figure 7 and Table S2).

Carotenoids-enriched extracts gave an overall pro-viability effect at all the tested concentrations for both types of pasta. With respect to HEK-293 buckwheat pasta extract gave a better rescue upon MMPP treatment for all the doses ranging from 50 ± 7% to 82 ± 5% compared to HEK-239 cells; extracts from egg pasta exerted a small rescue only at the lowest concentration of carotenoids 2 × 10^−6^ µM in the presence of MMPP, suggesting that the specific composition of extracts plays a key role in the response. Thus, buckwheat pasta showed a greater rescue capacity on the cell viability with respect to egg pasta and since the experiments were performed using the same amount of β-carotene, the difference could be due to the presence of minor compounds, such as flavonoids compounds, higher in the former.

Carotenoids species are involved in different chemical reactions exerting a dual nature of oxidant and anti-oxidant depending the biological context and their concentrations: this high reactivity is due to the presence of the conjugated double bounds [72]. Their antioxidant role was associated with the ability to delocalize the electrons given by oxidative species and radicals [73,74]. In the presence of high oxidative conditions their activity may be converted to pro-oxidant [75]. In our cases, this finding was observed in egg pasta for HEK-293 (both alone and in presence of MMPP) and MDCK cells in presence of MMPP, where different doses of acetone extract produced opposite effects.

### 3.5. Effect of Hydroalcoholic Extract on MMPP-Induced Cytotoxicity

**Figure 8:**
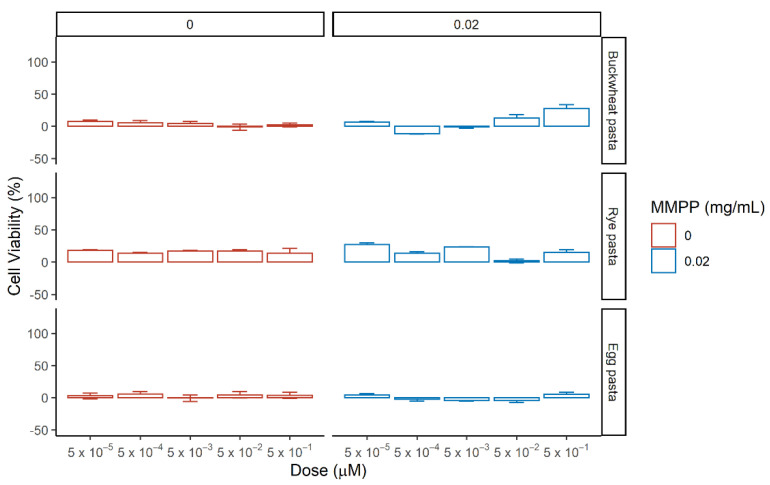
Effect of the pasta-derived hydroalcoholic extracts on the cell viability of HEK-293 in the absence (left, red) or presence (right, blue) of MMPP after 72 h incubation. Reported values correspond to mean of the difference in cell viability between tested phytocomplexes and reference (medium in red, and MMPP in blue) with standard error over three biological replicates. The percentage of cell viability was calculated using the ratio Abs_TEST_/Abs_CTRL_.

Afterwards, we evaluated the effects of the hydroalcoholic extracts from of buckwheat pasta, rye pasta and egg pasta on renal cells by testing different concentrations ranking from 5 × 10^−5^ µM to 5 × 10^−1^ µM with or without MMPP (Figure 8 and Table S3). The concentration used refers to the concentration of total phenolic content detected in the extract (Table 3). According to the content of the pasta samples reported in Table 1, for Buckwheat pasta the concentration of Indoleacetic acid was ranking from 1.85 × 10^−5^ µM to 1.85 × 10^−1^ µM, for caffeic acid derivates was ranking from 3.5 × 10^−6^ µM to 3.5 × 10^−2^ µM and for flavonoids content was ranking from 2.8 × 10^−5^ µM to 2.8 × 10^−1^ µM; for rye pasta the concentration of Indoleacetic acid was ranking from 2.2 × 10^−5^ µM to 2.2 × 10^−1^ µM, for caffeic acid derivates from 1.95 × 10^−5^ µM to 1.95 × 10^−1^ µM and for flavonoids species from 8.5 × 10^−6^ µM to 8.5 × 10^−2^ µM; for egg pasta the concentration of Indoleacetic acid was ranking from 4.25 × 10^−5^ µM to 4.25 × 10^−1^ µM, for flavonoids species from 7.5 × 10^−6^ µM to 1.85 × 10^−2^ µM while no caffeic acid derivatives were present.

Hydroalcoholic extract from egg pasta did not exert any significant effect on HEK-293 viability independently on the presence of oxidative stress. Extract from buckwheat pasta showed an enhancement of cell viability upon MMPP treatment only at the highest concentration (28 ± 6%). Rye pasta extract alone showed a general enhancement of the cell viability for all the doses ranging from 14 ± 7% to 18 ± 1% as well as in the presence of MMPP from 27 ± 3% to 13 ± 3%. The phytocomplex presented in the extracts, resulted to be more active than single standards, exerting an effect on cell viability at lower concentrations compared to the latter. These results suggest that in the hydroalcoholic extracts, the major role in the rescue could be attributed to the presence of caffeic acid derivatives, observing higher rescue for rye pasta and lower/negligible effect for egg past, in which they are not detectable.

**Figure 9:**
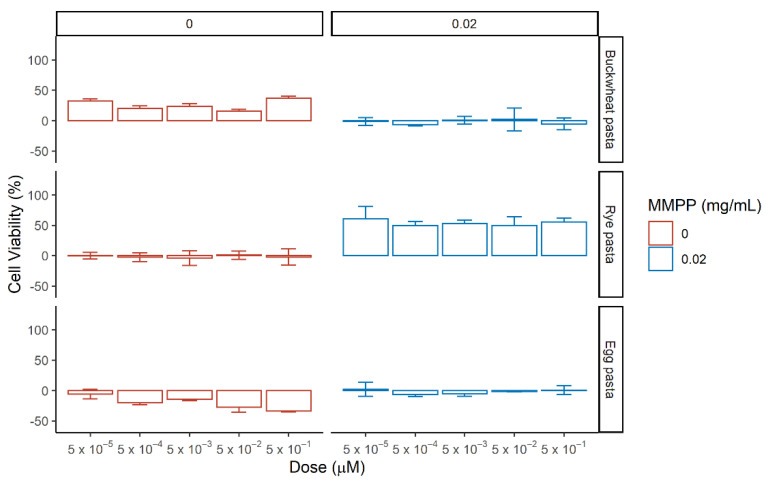
Effect of the pasta-derived hydroalcoholic extracts on the cell viability of MDCK in the absence (left, red) or presence (right, blue) of MMPP after 24 h incubation. Reported values correspond to mean of the difference in cell viability between tested phytocomplexes and reference (medium in red, and MMPP in blue) with standard error over three biological replicates. The percentage of cell viability was calculated using the ratio Abs_TEST_/Abs_CTRL_.

The hydroalcoholic extract were then tested on MDCK cells in the same conditions as before and the cell viability was assessed (Figure 9 and Table S4).

The hydroalcoholic extract from buckwheat and egg pasta displayed contrasting effects on cell viability: the first increases it, while the latter reduces it; no effect upon MMPP treatment was detected for both. Extract from rye pasta alone did not show any notable effect on cells viability, but it reveals a strong protective effect against MMPP-mediated oxidative stress at all the concentrations ranging from 50 ± 7% to 61 ± 21%. In standard conditions.

In the absence of MMPP, the high content of indoleacetic acid in the extract from egg pasta seems to exert a toxic effect on cell viability. Comparing the two cells models, we observed different behaviors with respect to the buckwheat and rye pasta in the absence of MMPP. In particular, HEK-293 showed an increase of viability with the latter, while MDCK with the former. The differences are related to the action of the phytocomplexes which are characterized by a higher content of flavonoids for the buckwheat pasta. The rescue on cell viability among samples, as observed for both cell lines, corresponds to the increase content of caffeic acid derivatives in rye pasta.

The study of the interaction between polyphenols containing phytocomplexes and renal cells, could help to investigate the known important effects on renal physiology exerted by those compounds [76], focusing on one of possible vehicle, pasta, typically associated with renal healthy diet. As a general rule, the metabolites of polyphenols have a plasma half-life of a few hours and are rapidly eliminated by the kidney and by the liver: the urinary and the biliary excretion route. The total amount of polyphenol metabolites excreted in urine is correlated not only with their maximum plasma concentration but also with their ability to be excreted by biliary rather than urinary tract. For example, urinary excretion of quercetin and its glycosides accounts for 0.3–1.4% of the ingested dose [77]; for caffeic acid and for ferulic acids accounts for 5.9% and 27% respectively. Therefore, pasta-derived polyphenols can have a positive impact on renal cells depending on their amount and metabolism. Further studies are needed to better define their contribution to kidney health.

## 4. Conclusions

The consumption of pasta is nowadays so diffused and accessible that this food could be one of the most common carriers for important metabolites, such as carotenoids and polyphenols that have been associated to general health and to protective mechanisms for kidney. In this study, we assessed the positive effects of bioactive compounds by comparing different type of pasta, highlighting how it represents an unconventional source of them. The comparison of the different cell responses between single molecules and extracts suggests considering the exerted activity as derived from the whole phytocomplex whit the presence of carotenoids such as lutein and polyphenols such as indoleacetic acid, respect to the individual species and the consequential attention to the raw materials. The scope of this study was (1) to strengthen the role of pasta as a food for the promotion of renal health in the healthy population and (2) for it to be a starting point for the improvement of the micronutrient profile in aproteic pastas. The results of this study suggest that the antioxidant species present in pasta play a major role in the protection of kidney cells from oxidative stress and reinforces the healthy role of quality organic pasta in the Mediterranean diet. High oxidative stress characterizes CKD and an anti-oxidant compounds can be beneficial to counteract it.

Even though the WHO and the FDA consider pasta as the most adequate food for fortification, in agreement with the very definition of fortified food (which is supplementing food with what is deficient in the population), we believe that pasta fortification is necessary only for special medical purpose, including protein-free pasta for CKD patients. For these patients, the micronutrient deficit in protein-free food and the necessary dietary restrictions to which they are subjected could generate nutritional deficiencies. In all the other cases, pasta fortification may not be necessary since pasta is naturally rich in complex carbohydrates and protein and low in fat and is therefore an already highly nutritious food by itself, especially if it is whole wheat based. Our study was meant to be an explorative preliminary study aimed at evaluating the possible beneficial effect that the polyphenolic components present in pasta could elicit on renal cells.

## Figures and Tables

**Table 1:** Caffeic derivatives (caffeic der), flavonoids and indoleacetic acid derivative (IAA der) content in cooked pasta samples. Data are the mean of three determinations (standard deviation < 5%). The percentage of single classes of identified compounds is shown in brackets.

	IAA der mg/100 g (%)	Flavonoids mg/100 g (%)	Caffeic der mg/100 g (%)
buckwheat pasta (1)	2.7 ± 0.086 (37)	4.1 ± 0.114 (56)	0.6 ± 0.025 (7)
rye pasta (2)	1.6 ± 0.041 (44)	0.6 ± 0.019 (17)	1.5 ± 0.052 (39)
Egg pasta (3)	4.8 ± 0.110 (85)	0.8 ± 0.021 (15)	-

**Table 2:** Carotenoids and lutein content in cooked pasta samples. Data are the mean of three determinations (standard deviation < 5%).

	Carotenoids mg/100 g	Lutein mg/100 g
buckwheat pasta (1)	0.08 ± 0.002	0.06 ± 0.002
Egg pasta (3)	0.16 ± 0.006	0.11 ± 0.004

**Table 3:** Total phenolic content (GAE, mg gallic acid/100g, Folin–Ciocalteu method) in cooked pasta samples. Data are the mean of three determinations (standard deviation < 5%).

	GAE, mg Gallic Acid/100 g Pasta
buckwheat pasta (1)	33.23 ± 0.598
rye pasta (2)	21.03 ± 0.273
egg pasta (3)	11.55 ± 0.1848

## Supplementary Materials

The following are available online at https://www.mdpi.com/article/10.3390/nu13041131/s1, Table S1: Nutritional Table. Table S2—Detection of cell viability variation of HEK-293 and MDCK incubated with increasing amount of carotenoid-enriched fraction from Buckwheat pasta and Egg pasta on medium and MMPP for 72 (HEK-293) and 24 (MDCK) hours. * *p* < 0.5, ** *p* < 0.01, *** *p* < 0.001. Table S3—Detection of cell viability variation of HEK-293 incubated with increasing amount of hydroalcoholic extract from Buckwheat pasta, Rye pasta and Egg pasta on medium and MMPP for 72 h. * *p* < 0.5, ** *p* < 0.01, *** *p* < 0.001. Table S4—Detection of cell viability variation of MDCK incubated with increasing amount of hydroalcoholic extract from Buckwheat pasta, Rye pasta and Egg pasta on medium and MMPP for 24 h. * *p* < 0.5, ** *p* < 0.01, *** *p* < 0.001. Figure S1: MS spectra of Kaempferol in Buckwheat cooked pasta hydroalcoholic extract. Figure S2: MS spectra of quercetin in Buckwheat cooked pasta hydroalcoholic extract. Figure S3: MS spectra of rutin in cooked pasta hydroalcoholic extract. Figure S4: MS spectra of ferulic acid one in rye cooked pasta hydroalcoholic extract. Figure S5: MS spectra of apigenin diglicosides in egg cooked pasta hydroalcoholic extract. Figure S6: Detection cell viability of HEK-293 incubated with increasing amount of Indoleacetic acid, β-carotene and caffeic acid for 24 (A) and 48 (B) hours Reported values correspond to mean of cell viability with standard error over three biological replicates. The percentage of cell viability was calculated using the ratio Abs_TEST_/Abs_CTRL_.
