# Supplementary material for: Preliminary Study on Pasta Samples Characterized in Antioxidant Compounds and Their Biological Activity on Kidney Cells
Source: Nutrients. 2021 Mar 30;13(4):1131. doi: 10.3390/nu13041131 (PMC8067028; doi:10.3390/nu13041131)
Supplement: Supplementary file 1 [file nutrients-13-01131-s001.pdf]

SUPPLEMENTARY MATERIALS

Table S1 – Nutritional Table

	Buckwheat pasta	Rye pasta	Egg pasta
Kcal/100g	356	342	270
Carbohydrates (g/100g)	70.3	71.1	75.3
Sugars (g/100g)	3.7	2.47	1.4
Protein (g/100g)	11.2	7.6	15.9
Fats (g/100g)	1.9	1.5	4.1
Saturated (g/100g)	0.5	0.4	1.2
Mono (g/100g)	0.4	/	1.8
Poly (g/100g)	1	/	1.1
Fibers (g/100g)	7.2	6.9	3.5
Resistant Starch (g/100g)	1.2	1	1.3
Soluble Starch (g/100g)	61.1	54	60.2
Total Starch (g/100g)	62.3	55	61.5

Table S2 – Detection of cell viability variation of HEK-293 and MDCK incubated with increasing amount of carotenoid-enriched fraction from Buckwheat pasta and Egg pasta on medium and MMPP for 72 (HEK-293) and 24 (MDCK) hours. * p<0.5, ** p<0.01, ***p<0.001

Cell	Extract	Dose (uM)	Mean Effect (%)	SE (%)	*p*
HEK - 293	Buckwheat pasta	2e-06	12	8	
		2e-05	11	4	
		2e-04	13	2	
		2e-03	17	2	*
	Buckwheat pasta+ MMPP (0.02 mg/mL)	2e-06	32	6	
		2e-05	30	3	**
		2e-04	21	9	
		2e-03	3	2	
	Egg pasta	2e-06	11	5	
		2e-05	4	3	
		2e-04	-28	1	
		2e-03	-29.0	0.4	
	Egg pasta + MMPP (0.02 mg/mL)	2e-06	10	10	
		2e-05	0	1	
		2e-04	-28	3	
		2e-03	-21.6	0.1	
MDCK	Buckwheat pasta	2e-06	27	19	
		2e-05	19	11	
		2e-04	22	2	**
		2e-03	-3	6	

Buckwheat pasta + MMPP (0.02 mg/mL)	2e-06	76	7	***
	2e-05	54	4	*
	2e-04	50	7	*
	2e-03	83	6	***
Egg pasta	2e-06	19	3	*
	2e-05	19	3	*
	2e-04	3	2	
	2e-03	14	1	***
Egg pasta + MMPP (0.02 mg/mL)	2e-06	8	2	*
	2e-05	3	1	
	2e-04	-11	2	*
	2e-03	-18	1	***

Table S3 – Detection of cell viability variation of HEK-293 incubated with increasing amount of hydroalcoholic extract from Buckwheat pasta, Rye pasta and Egg pasta on medium and MMPP for 72 hours. * p<0.5, ** p<0.01, ***p<0.001

Cell	Extract	Dose (uM)	Mean Effect (%)	SE (%)	*p*
HEK-293	Buckwheat pasta	5e-05	8	2	
		5e-04	6	3	
		5e-03	4	3	
		5e-02	-1	5	
		5e-01	2	3	
	Buckwheat pasta + MMPP (0.02 mg/mL)	5e-05	7	1	
		5e-04	-12	1	
		5e-03	-1	1	
		5e-02	13	5	
		5e-01	28	6	
	Rye pasta	5e-05	18	1	*
		5e-04	14	1	
		5e-03	17	1	*
		5e-02	18	2	*
		5e-01	14	7	
	Rye pasta + MMPP (0.02 mg/mL)	5e-05	27	3	*
		5e-04	13	3	
		5e-03	23	1	*
		5e-02	18	3	
		5e-01	15	4	
	Egg pasta	5e-05	3	4	
		5e-04	6	4	
		5e-03	0	5	
		5e-02	5	5	
		5e-01	4	5	
	Egg pasta + MMPP (0.02 mg/mL)	5e-05	4	2	
		5e-04	-3	2	
		5e-03	-4	1	
		5e-02	-4	3	
		5e-01	6	3	

Table S4 – Detection of cell viability variation of MDCK incubated with increasing amount of hydroalcoholic extract from Buckwheat pasta, Rye pasta and Egg pasta on medium and MMPP for 24 hours. * p<0.5, ** p<0.01, ***p<0.001

Cell	Extract	Dose (uM)	Mean Effect (%)	SE (%)	*p*
MDCK	Buckwheat pasta	5e-05	32	3	*
		5e-04	21	4	*
		5e-03	24	5	***
		5e-02	16	3	
		5e-01	37	4	***
	Buckwheat pasta + MMPP (0.02 mg/mL)	5e-05	-2	7	
		5e-04	-6	2	
		5e-03	1	7	
		5e-02	2	19	
		5e-01	-5	10	
	Rye pasta	5e-05	0	5	
		5e-04	-2	7	
		5e-03	-4	12	
		5e-02	1	7	
		5e-01	-3	13	
	Rye pasta + MMPP (0.02 mg/mL)	5e-05	61	21	**
		5e-04	50	7	*
		5e-03	53	5	
		5e-02	50	14	
		5e-01	56	6	**
	Egg pasta	5e-05	-6	8	
		5e-04	-20	3	*
		5e-03	-14	2	
		5e-02	-27	8	
		5e-01	-34	1	***
	Egg pasta + MMPP (0.02 mg/mL)	5e-05	2	12	
		5e-04	-6	4	
		5e-03	-5	4	
		5e-02	-1	1	
		5e-01	1	7	

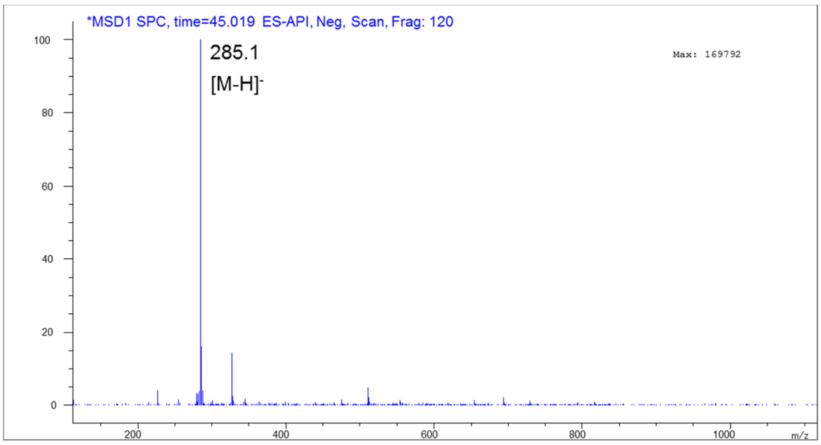

Figure S1 - MS spectra of Kaempferol in Buckwheat cooked pasta hydroalcoholic extract

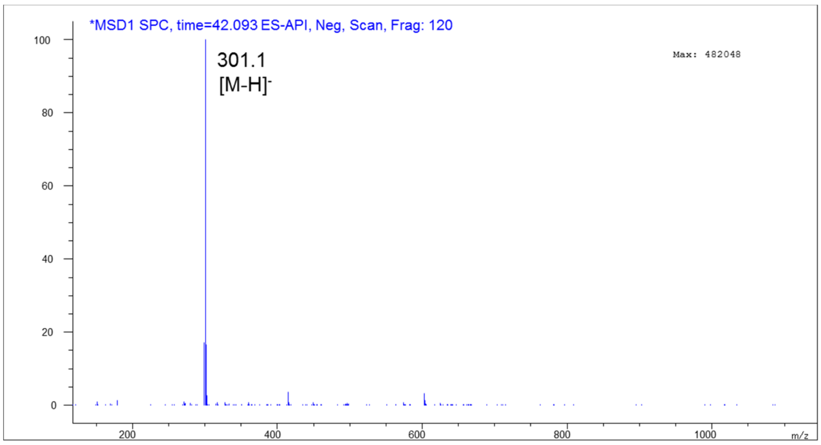

Figure S2 - MS spectra of quercetin in Buckwheat cooked pasta hydroalcoholic extract

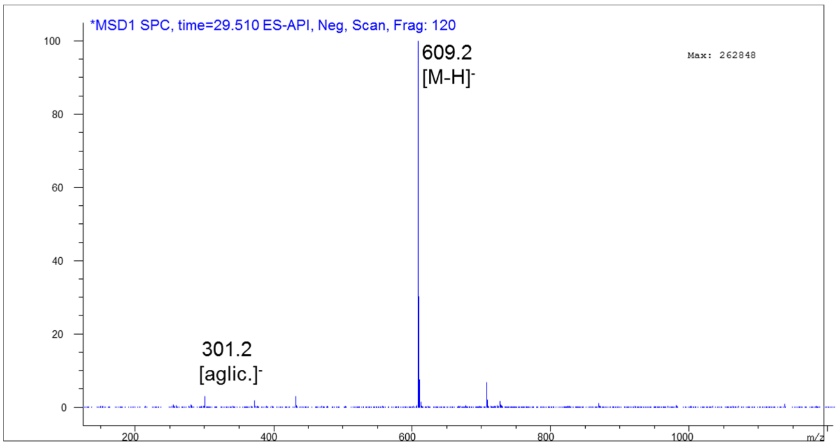

Figure S3 - MS spectra of rutin in cooked pasta hydroalcoholic extract

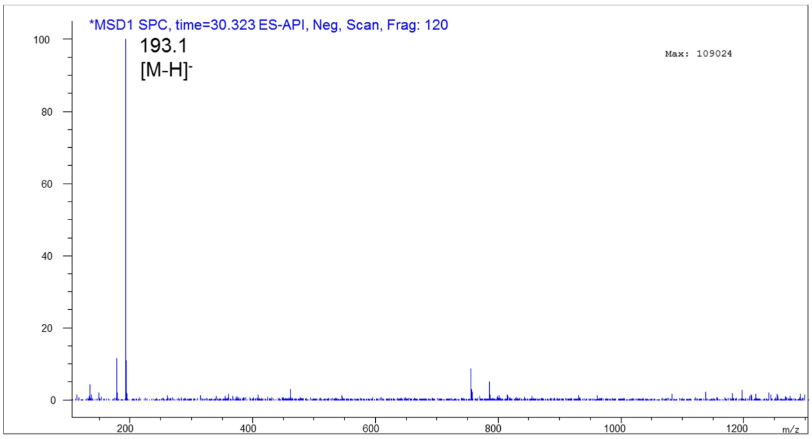

Figure S4 - MS spectra of ferulic acid one in rye cooked pasta hydroalcoholic extract

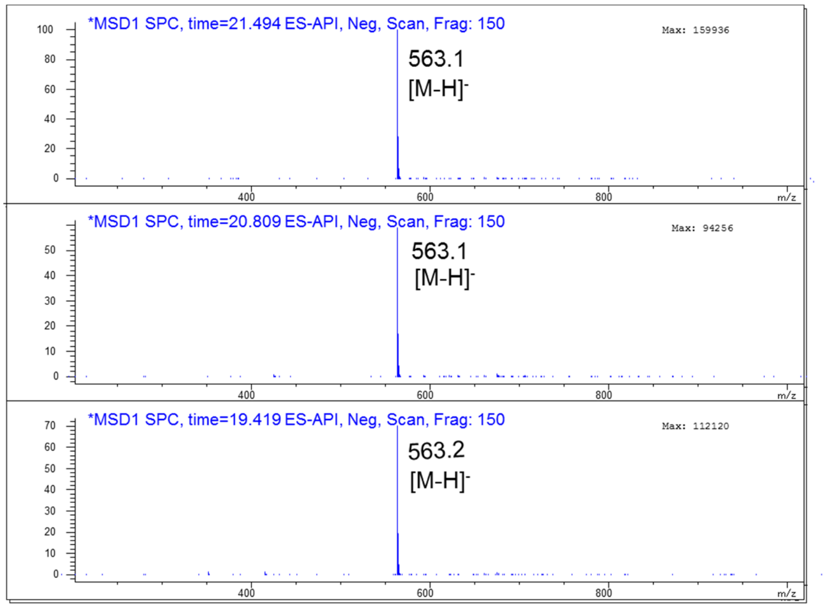

Figure S5 - MS spectra of apigenin diglicosides in egg cooked pasta hydroalcoholic extract

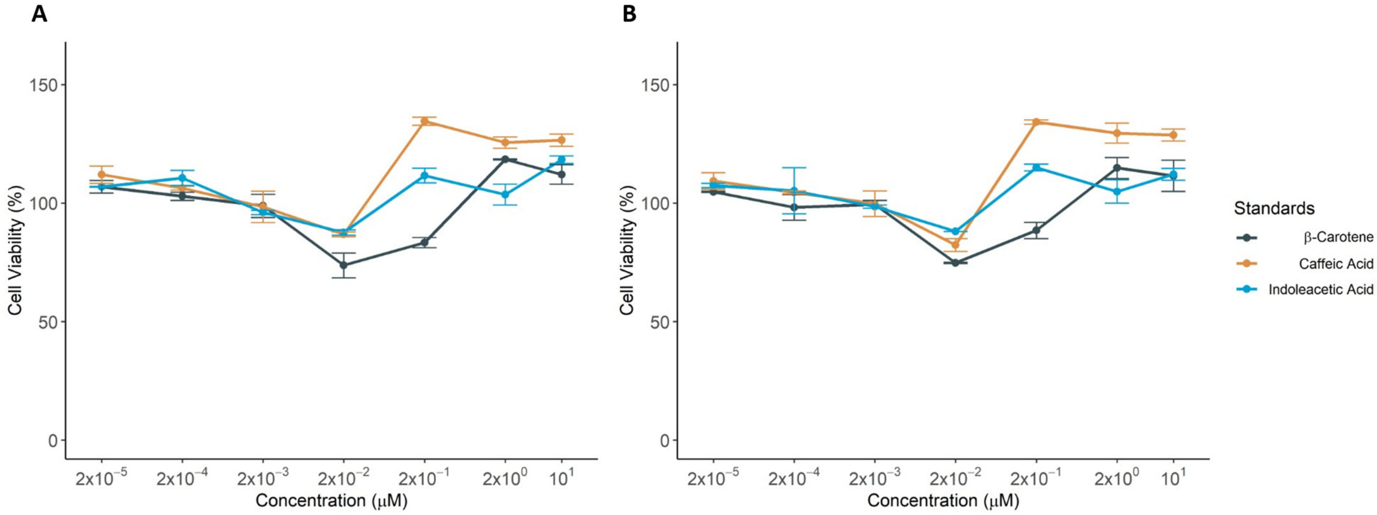

Figure S6 - Detection cell viability of HEK-293 incubated with increasing amount of Indoleacetic acid, β-carotene and caffeic acid for 24 (A) and 48 (B) hours. Reported values correspond to mean

of cell viability with standard error over three biological replicates. The percentage of cell viability was calculated using the ratio Abs_{TEST}/Abs_{CTRL}.
